# Inhibition of Nigral Microglial Activation Reduces Age-Related Loss of Dopaminergic Neurons and Motor Deficits

**DOI:** 10.3390/cells11030481

**Published:** 2022-01-30

**Authors:** Tzu-Feng Wang, Shih-Ying Wu, Bo-Syong Pan, Sheng-Feng Tsai, Yu-Min Kuo

**Affiliations:** 1 Taiwan International Graduate Program in Interdisciplinary Neuroscience, National Cheng Kung University and Academia Sinica, Taipei 11529, Taiwan; wangtft@gmail.com; 2 Department of Cell Biology and Anatomy, College of Medicine, National Cheng Kung University, Tainan 70101, Taiwan; eric04142000@hotmail.com; 3 Institute of Basic Medical Sciences, College of Medicine, National Cheng Kung University, Tainan 70101, Taiwan; bi30rd92@hotmail.com (S.-Y.W.); bearpan310@yahoo.com.tw (B.-S.P.); 4 Department of Cancer Biology, Wake Forest Baptist Medical Center, Wake Forest University, Winston Salem, NC 27157, USA

**Keywords:** microglial activation, dopaminergic neurons, exercise, brain-derived neurotrophic factor

## Abstract

Parkinson’s disease (PD) is an age-related neurodegenerative disease caused by a selective loss of dopaminergic (DA) neurons in the substantia nigra (SN). Microglial activation is implicated in the pathogenesis of PD. This study aimed to characterize the role of microglial activation in aging-related nigral DA neuron loss and motor deficits in mice. We showed that, compared to 3-month-old mice, the number of DA neurons in the SN and the expression of dopamine transporter (DAT) in the striatum decreased during the period of 9 to 12 months of age. Motor deficits and microglial activation in the SN were also evident during these months. The number of DA neurons was negatively correlated with the degrees of microglial activation. The inhibition of age-related microglial activation by ibuprofen during these 3 months decreased DA neuron loss in the SN. Eliminating the microglia prevented systemic inflammation-induced DA neuron death. Forcing mice to run during these 3 months inhibited microglial activation and DA neuron loss. Blocking the brain-derived neurotrophic factor (BDNF) signaling eliminated the exercise-induced protective effects. In conclusion, nigral DA neurons were susceptible to local microglial activation. Running exercise upregulated BDNF-TrkB signaling and inhibited microglial activation during aging. Long-term exercise can be considered as a non-pharmacological strategy to ameliorate microglial activation and related neurodegeneration.

## 1. Introduction

Pathologically, Parkinson’s disease (PD) is characterized by a chronic and selective loss of DA neurons in the substantia nigra (SN). The precise mechanism for dopaminergic (DA) neuron loss in the SN is unclear. In addition to aging, the most important risk factor for idiopathic PD [1], inflammation is closely related to DA neuron death in the SN [2,3,4]. The levels of pro-inflammatory cytokines are elevated in the cerebrospinal fluid, serum, striatum, and SN of PD patients [4]. Using a positron emission tomography scan with radiotracers for activated microglia and dopamine transporter (DAT), a negative correlation between these two markers in the DA nigrostriatal system has been reported in early PD patients [5]. These findings suggest that microglial activation and neuroinflammation are intimately associated with the survival of DA neurons in the SN.

Microglial activation is a common phenomenon in multiple neurodegenerative diseases [6,7]. Microglia are innate immune cells that patrol their surroundings by thin processes extending from the cell body, to maintain homeostasis of the central nervous system (CNS) under physiological conditions. Upon exposure to stimulatory signals, microglia will transform (so-called activation), including changes in morphology, gene expression, secretion content, and behavior, in order to execute their innate immune function [8]. It has been shown that systemic inflammation can be transduced to the CNS through inflammatory mediators, which trigger microglial activation. Microglia with a prior experience of activation are susceptible to meeting subsequent stimulation with exaggerated responses, which contributes to the pathogenesis of age-related neurodegenerative diseases [6,7,9,10].

Regular physical exercise is beneficial to many aspects of health, including brain function. Exercise is known to enhance adult hippocampal neurogenesis and neuroplasticity, improve learning and memory, delay cognitive decline, and inhibit neuroinflammation [11,12,13,14,15,16,17,18,19,20,21,22]. In 6-hydroxydopamine and 1-methyl-4-phenyl-1,2,3,6-tetrahydropyridine (MPTP) PD rodent models, exercise enhances functional recovery and attenuates the loss of striatal dopamine [15,23,24,25]. Although the underlying mechanism remains unclear, neurotrophic factors such as brain-derived neurotrophic factor (BDNF) have been implicated in the beneficial effects of exercise [12,26]. BDNF is mainly synthesized in the CNS [27] and plays a key role in the development of the nervous system [28,29]. It has been shown that patients with neurodegenerative diseases had reduced levels of BDNF in their brains [30,31]. Increased expressions of BDNF after exercise have been demonstrated in various PD rodent models [32,33,34]. The exercise-induced upregulation of BDNF has been suggested as a promising therapeutic agent for PD [35].

This study aimed to characterize the role of microglial activation in progressive DA neuron loss in the SN of mice during aging. Initially, we characterized the changes of microglial activation and the number of DA neurons in the SN of different ages of male C57BL/6 mice. By using pharmacological and exercise-based approaches, the role of microglial activation and related neurotoxicity were examined in the SN and striatum of mice. The role of the BDNF-tropomyosin receptor kinase (TrkB) signaling pathway in the exercise-induced regulation of microglial activation was also investigated. 

## 2. Materials and Methods

### 2.1. Animals

Animal experiments were approved by the National Cheng Kung University Institutional Animal Care and Use Committee (approval number 101065) and were in accordance with local and national guidelines. Male C57BL/6J mice (at 3, 6, 9, and 12 months old) were obtained from the National Laboratory Animal Center, Tainan Facility (https://www.nlac.narl.org.tw/eng/index.asp, accessed on 1 November 2021), accredited by the AAALAC. The mice were housed, with 4 to 5 per cage, at the National Cheng Kung University Laboratory Animal Center with a stable temperature (24 ± 1 °C), a 12-h light/dark cycle (light on at 7 a.m.), and unlimited access to food and water. The housing conditions and animal health were monitored by the Laboratory Animal Center, NCKU.

### 2.2. Treadmill Running Training

Mice were randomly divided into three groups: (1) 12-month-old sedentary (Sed) mice; (2) 12-month-old mice that had undergone 1 month of treadmill running (TR), beginning when they were 11 months old (TR(1Mo)); (3) 12-month-old mice that had undergone 3 months of treadmill running beginning when they were 9 months old (TR(3Mo)). The detailed protocol for TR has been described elsewhere [12]. During the familiarization phase, all the TR mice were trained for 10 min/day for 5 days to run on a level, motor-driven treadmill (T510E, Diagnostic and Research Instruments Co., Taoyuan, Taiwan) to reduce environmental and handling stress. For the Sed and TR(3Mo) groups, the familiarization phase began 1 week before they were 9 months old, and the running speed was set at 9 m/min; for the TR(1Mo) group, it began 1 week before they were 11 months old and the running speed was set at 8 m/min. Following the familiarization week, TR mice were run at a speed 1 m/min faster than that used in the familiarization phase for 20–60 min/day (an increment of 10 min/day), 5 days for the first week, followed by 60 min/day at the same speed, 5 days/week for the next 3 weeks. For the TR(3Mo) group, the speed was increased by 1 m/min every month. The running speed fulfilled the intensity criterion of mild exercise. The training was from 5 p.m. to 6 p.m., the last hour of the light cycle, to harmonize with the circadian activity of the mice. Each mouse was assigned to a fixed lane during the training program, to minimize the confounder of novelty. There were a few occasions on which mice were excluded from the study because they were not compliant with the training (e.g., they refused to run or they kept jumping out of their running lane). Sed mice were placed on an immobile treadmill next to the TR treadmill for the training period. One day after the conclusion of TR, the mice were sacrificed by an overdose of isoflurane, and the brains were removed and processed as described in Section 2.7.

### 2.3. Ibuprofen Supplement

Ibuprofen (I4883, Sigma-Aldrich, St. Louis, MO, USA) was freshly dissolved in drinking water (10 mg/mL), which was provided daily to 9-month-old mice for 3 months. Control mice were given regular drinking water only.

### 2.4. Ki20227 and Lipopolysaccharide Administration

Ki20227, a selective colony-stimulating factor 1 receptor (also known as macrophage colony-stimulating factor receptor and CD115) inhibitor, blocks macrophage differentiation and inhibits microglial viability [36]. One microliter of Ki20227 (100 μg/μL, in 0.1% DMSO, 4481, R&D Systems, Minneapolis, MN, USA) was injected into the right SN (stereotaxic coordinates in mm from the bregma: anterior/posterior, −3.0; lateral, −1.2; ventral, −4.4) of 3-month-old mice. An equal volume of saline was injected into the left SN to create an internal sham control. The infusion was controlled using a syringe pump at a rate of 0.1 μL/min. The needle was removed 20 min after the infusion was completed. The next day, mice were intraperitoneally (i.p.) injected with 1 mg/kg of lipopolysaccharide (LPS) (L2880, *E. coli* 055:B5 strain Sigma-Aldrich, St. Louis, MO, USA) to induce peripheral inflammatory and microglial activation [34]. Mice were sacrificed 3 days later by an overdose of isoflurane. 

### 2.5. Beam Traversal Test

The beam traversal test was performed as described [37]. The mice were trained to traverse the length of a 1-meter acrylic beam consisting of four 25-centimeter sections decreasing in width from 3.5 cm to 0.5 cm in 1-centimeter decrements. Each mouse was trained for 2 days before the test. On the test day, a mesh grid (squares of 1 cm) of the corresponding width was placed 1 cm above the beam surface. The mouse was put on the mesh grid and was video-recorded while it was traversing the grid-surfaced beam. Each mouse underwent five trials. The time taken to traverse the beam and the number of erroneous steps (e.g., a paw slipping through the mesh grid or placed on the side rather than the top of the grid) were quantified and averaged over five trials for each mouse. The behavioral recordings were analyzed by another researcher who was not the executor of the beam traversal test.

### 2.6. Rotarod Test

An accelerating rotarod (Singa Technology Corp., Taipei, Taiwan) was utilized to determine the motor coordination of another set of mice. The rotarod consisted of a rotating spindle of 5 cm in diameter. Each mouse was trained for 2 days before the test. The mouse was placed on the rotating rod in a separate compartment at a speed of 5 rpm for an accumulated time of 3 min. On the test day, the rotarod speed was set to increase from 5 rpm to 40 rpm after 3 min. The tendency to fall off was automatically recorded by magnetic trip plates. The trial was stopped if the mouse fell off or if it stayed on for a maximum of 3 min.

### 2.7. Preparing Brain Tissue

The mice were anesthetized with an overdose of isoflurane and perfused ice-cold 0.1 M phosphate-buffered saline (PBS) into the left cardiac ventricle before the removal of their brains. The left hemisphere was dissected to collect the SN and striatum, frozen in liquid nitrogen, and stored at –80 °C until further processing. The right hemisphere was fixed in 4% paraformaldehyde at 4 °C for 48 h. The brain samples were then dehydrated in serial graded sucrose solutions (10%, 20%, 30%, and 35%, in 0.1 M phosphate buffer, pH 7.4) for a week in total and then embedded into FSC 22 frozen section media (3801480, Leica Biosystems, Nussloch, Germany). The brains were sequentially sliced into 30-micrometer coronal sections for immunohistochemistry and stored in cryoprotectant at –20 °C until they were used.

### 2.8. Immunohistochemistry

The free-floating 30-micrometer brain sections of interest were selected, washed with 0.3% Triton X-100 (X100, Sigma-Aldrich, St. Louis, MO, USA) in PBS, immersed in 3% H_2_O_2_ to abolish endogenous peroxidase activity, and blocked with 3% normal goat serum (Cat# 005-000-001, RRID: AB_2336983, Jackson ImmunoResearch Inc., West Grove, PA, USA) to reduce non-specific bindings of antibody for 1 h at room temperature. The sections were then incubated with rabbit anti-tyrosine hydroxylase (TH) (1:2000; MAB152, Millipore, Darmstadt, Germany) for DA neurons, rabbit anti-ionized calcium-binding adapter molecule-1 (Iba1) (1:2000; 019-19741, Wako Pure Chemical Industries, Osaka, Japan) for microglia, and goat anti-DAT (1:1000; SC-1433, Santa Cruz Biotechnology, Dallas, TX, USA) for DA neuron terminals. Sections were then incubated with appropriate biotin-conjugated secondary antibodies and avidin-biotin-peroxidase (PK-6100, Vectastain Elite ABC Kit, Vector Laboratories, Burlingame, CA, USA) using diaminobenzidine to visualize signals. In some cases, signals were enhanced using nickel ammonium sulfate [38]. The signals were evaluated based on the morphologies and intensities after subtracting the signals of the primary antibody-omitted negative controls. Tissue was mounted onto silane-coated slides (5116, Microslides, MUTO Pure Chemicals, Tokyo, Japan), allowed to dry overnight. The stained sections were dehydrated in graded ethanol with increasing concentrations and coverslipped with Micromount (3801731, Leica Biosystems, Nussloch, Germany).

### 2.9. Counting Cells

Most of the TH^+^ neurons in the central nervous system are located in the SN and the ventral tegmental area (VTA) of the ventral midbrain. We followed the criteria that have been described elsewhere [39,40], to separate these two regions. The entire SN, from 2.5 to 3.9 mm posterior to the bregma [41], was collected with an average of 40 coronal sections. The numbers of TH^+^ and Iba1^+^ cells were counted in every 6th section in the right SN, based on the principles of design-based stereology [42,43,44,45]. The two investigators who performed the cell counting, using different sets of non-repeating sections, were blinded to the experimental groups. The SN was first outlined with the 10× objective lens of a Zeiss microscope (Carl Zeiss, Oberkochen, Germany) and the number of positively stained cells was counted using a computer-controlled x-y-z motorized stage and 40× objective lens. The number of labeled cells per brain slide was divided by the slide selection ratio (6/40) to obtain the total number of cells in each SN.

### 2.10. Quantifying the Microglial Area

The areas of Iba1^+^ cells were measured in a fixed area or at every 6th section of the entire SN. Micrographs were taken with a Zeiss Axio microscope attached to a digital camera (Axiocam 305 color, Carl Zeiss, Oberkochen, Germany) controlled by a computer equipped with imaging software (Axiovision v 4.8, Carl Zeiss, Oberkochen, Germany). The Iba1^+^ areas were obtained using ImageJ software (version 1.51 w, NIH, Bethesda, MD, USA) by measuring the areas with Iba1^+^ intensities higher than a given background threshold. The background intensity threshold was fixed and used for all sections.

### 2.11. Quantifying DA Fibers in the Striatum

The striatal DA fibers were evaluated by quantitating the optical density of immunostained DAT^+^ signals in the striatum, using ImageJ software (version 1.51 w, NIH, Bethesda, MD, USA), by an investigator blind to the experimental groups. We only collected the central part of the striatum (1.54 mm anterior to and 0.94 mm posterior to the bregma [41]), which consisted of an average of 80 30-micrometer sections. The images of the DAT immunostained striatum were captured in every 12th section of the right striatum with a 2.5× objective lens. The same capture settings were applied for all sections. The optical density of the cortex was taken as a background and subtracted from the value of the DAT^+^ signal from the striatum. The average of the sections was calculated as the optical density in each striatum.

### 2.12. Transmission Electron Microscopy

Mice (6 and 12 months old) were anesthetized with an overdose of isoflurane and perfused from the left ventricle with 0.9% normal saline for 2 min, and then with ice-cold fixative buffer (4% paraformaldehyde and 1% glutaraldehyde in PBS, pH 7.6) for 10 min. The SN was quickly dissected out from the brain and placed in 2.5% buffered glutaraldehyde at 4 °C. Twenty-four hours later, the SN was embedded in 2% agarose, chilled at 4 °C for 30 min, and cut into 50-micrometer sections with a vibratome. After removing the agarose, the sections were incubated in 1% osmium tetroxide for 1 h. For immunostaining electron microscopy (immuno-EM), the 50-micrometer section was first immunostained with TH (1:500) (Millipore, Darmstadt, Germany) antibodies before being incubated in 1% osmium tetroxide. The stained sections were dehydrated in graded ethanol and embedded in epoxy resin at 60 °C for 24 h. The sample block was cut into 1-micrometer ultrathin sections using an ultramicrotome (Leica Reichert Ultracut, Leica Microsystems, Wetzlar, Germany) and then stained with uranyl acetate and lead citrate [46]. The stained section was viewed under a transmission electron microscope (JEM-1400, JEOL Ltd., Tokyo, Japan).

### 2.13. Western Blotting

The SNs were homogenized (1:1, weight: volume in brain tissue) in the RIPA buffer (1% NP40, 10 μg/mL aprotinin, 1 μg/mL leupeptin, 1 mM phenylmethanesulfonyl fluoride, 0.5 mM sodium vanadate, 137 mM NaCl, 20 mM Tris-HCl (pH 8.0)) containing a mixture of protease inhibitors (Complete Mini Protease Inhibitor Cocktail Tablets; Roche Diagnostics, Basel, Switzerland) and then centrifuged at 13,000× *g* at 4 °C for 30 min. The protein concentrations of the supernatants were adjusted to the same concentration. Supernatants (30 μg of total protein) were mixed with sample buffer containing 0.5 M of dithiothreitol, heated to 80 °C for 10 min, loaded into each well of 4–12% polyacrylamide gel (NP0321PK2, NuPAGE™, Thermo Fisher Scientific, Waltham, MA USA) and resolved at 120 V for 2 h. The separated proteins were transferred to a polyvinylidene fluoride membrane (1620117, Bio-Rad Laboratories, Hercules, CA, USA), blocked in 5% nonfat milk for an hour, and hybridized with primary antibodies: BDNF (1:1000; SC-33905, Santa Cruz Biotechnology, Dallas, TX, USA), TrkB (1:3000; BD610102, BD Biosciences, San Jose, CA, USA), NF-κB p65 (1:20,000; 8242 S, Cell Signaling, Danvers, MA, USA), Phospho-NF-κB p65 (1:20,000; 3036 S, Cell Signaling, Danvers, MA, USA), and β-actin (1:40,000; MAB1501, Millipore, Darmstadt, Germany). The bound antibodies were detected using an enhanced chemiluminescence substrate (NEL103E001EA, PerkinElmer, Waltham, MA, USA). The band densities were measured using an imaging system (BioChemi System, UVP, Upland, CA, USA) and analyzed with ImageJ software (version 1.51 w, NIH, Bethesda, MD, USA). For gel loading control, membranes were re-probed with monoclonal β-actin antibody (1:40,000; Millipore, Darmstadt, Germany).

### 2.14. Quantifying TNF and IL-6 in the SN

Mouse TNF (BMS607-3, Thermo Fisher Scientific, Waltham, MA, USA) and IL-6 (KMC0061, Thermo Fisher Scientific, Waltham, MA, USA) ELISA kits were used to quantify the levels of TNF and IL-6 in the mouse brain supernatants. The absorbance of each specimen was read at 450 nm in the spectrophotometer. Standard curves were obtained from known concentrations of TNF and IL-6, as provided by the kits.

### 2.15. Preparing TrkB shRNA and Gene-Expressing Lentivirus

The lentivirus production was performed as previously described [43]. Three sets of short hairpin (sh) RNA (shTrkB and shLacZ)-expressing lentiviral plasmids (pLKO.1-puro) were obtained from the National RNAi Core Facility of Academia Sinica, Taipei, Taiwan. The effectiveness of the shTrkB was validated by transfecting the shTrkB- and shLacZ-expressing plasmids into mouse neuroblastoma N2a cells and then using Western blotting to quantify the TrkB level. Transfected cells expressing the lowest level of TrkB protein were selected. The selected encoding sequences of shRNA against TrkB and LacZ were 5′-CAGCAACCTGCGGCACATAAA-3′ and 5′-TGTTCGCATTATCCGAACCAT-3′, respectively. The lentivirus was packaged by transiently co-transfecting shRNA expression plasmid, psPAX2 packaging plasmid, and pMD2G envelope plasmid into HEK293T cells. The medium was collected 48 h after post-transfection, and the debris was removed using low-speed centrifugation. High-titer stocks were prepared with ultracentrifugation at 100,000× *g* at 4 °C for 90 min. Viral pellets were suspended in a serum-free medium and stored at −70 °C.

### 2.16. Delivering shTrkB to the Striatum

To block the TR-related BDNF-TrkB signaling pathway, 9-month-old mice were injected with shTrkB or shLacZ viruses into the striatum one week before the TR familiarization phase. Viruses injected into the striatum can be retrogradely transported to nigral DA neurons through the nigrostriatal pathway, which avoids injuring the SN [47]. The shTrkB viral solution (1 μL) was injected into the right striatum (stereotaxic coordinates in mm from the bregma: anterior/posterior, +0.14; lateral, −2.0; ventral, −3.5) at a flow rate of 0.1 μL /min by a syringe pump. The needle was removed 10 min after the infusion was completed. The shLacZ viral solution (1 μL) was injected into the left striatum (stereotaxic coordinates in mm from the bregma: anterior/posterior, +0.14; lateral, +2.0; ventral, −3.5) and was the internal sham control. The mice were given two more shTrkB/shLacZ injections; one when they were 10-month-old and one when they were 11-month-old, to ensure that TrkB expression would be inhibited.

### 2.17. Statistical Analysis

Data are presented as mean ± standard deviation. Significance was set at *p* < 0.05. The number of TH^+^ cells, the intensity of DAT^+^ signals, area of Iba1^+^ signals, number of Iba1^+^ cells, scores of behavior tests, concentrations of cytokines, and levels of BDNF and TrkB in different TR groups were analyzed using one-way analysis of variance (ANOVA) followed by Bonferroni post hoc tests if the overall effect was significant. The associations between DA neurons and microglial activation were evaluated using the Pearson correlation. Unaired two-tailed Student’s *t*-tests were used to analyze the effects of ibuprofen supplementation. Paired two-tailed Student’s *t*-tests were used to analyze the effects of Ki20227 and LPS administration. Two-way ANOVA was used to analyze the effects TR(3Mo) and shTrkB and any possible interaction between them. Bonferroni post hoc tests were used if the main effects or interactions were significant. Statistical analyses were performed using the GraphPad Prism (version 7.04 for Windows, GraphPad Software, San Diego, CA, USA). The numbers of mice used in each experiment are shown in Table S1. Details of statistics results are shown in Table S2.

## 3. Results

### 3.1. Age-Related DA Neuron Loss in the SN Is Associated with Microglial Activation

**Figure 1:**
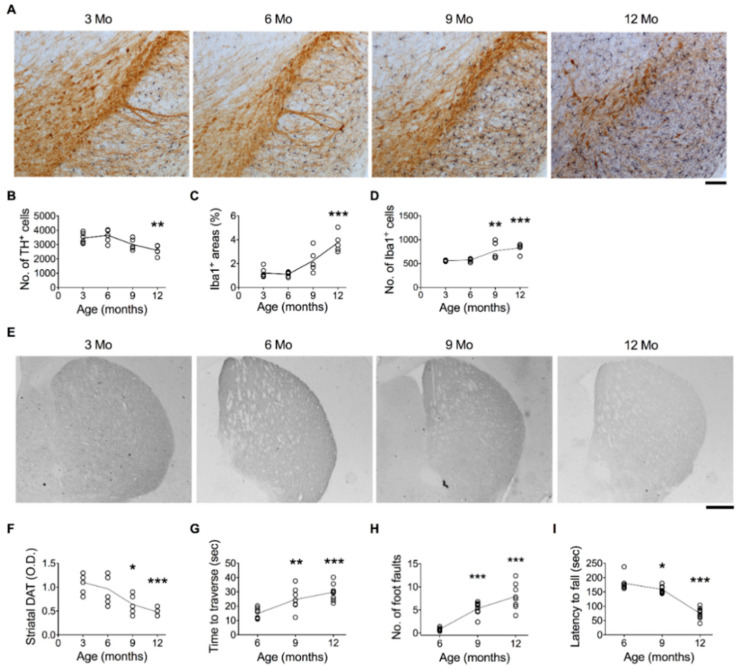
Temporal profiles of the number of DA neurons and degrees of microglial activation in the SN, DAT^+^ signals in the striatum, and motor performance. (**A**) Representative dual immunohistochemical images of the TH^+^ neurons (brown) and Iba1^+^ (purple-blue) cells in the SNs of 3-, 6-, 9-, and 12-month-old mice. Bar = 100 μm. (**B–D**) Quantitative results of the number of TH^+^ neurons, Iba1^+^ areas, and numbers of Iba1^+^ cells. (**E**) Representative immunohistochemical images show DAT^+^ signals in the striata of 3-, 6-, 9-, and 12-month-old mice. Bar = 500 μm. (**F**) Quantitative results of DAT^+^ signals. (**G**) Time to traverse the beam. (**H**) The number of foot faults made while traversing the beam. (**I**) Tendency to fall off the rotarod. * *p* < 0.05, ** *p* < 0.01, *** *p* < 0.001 versus 3-month-old mice, one-way ANOVA, Bonferroni’s post hoc test. The numbers of mice used in the experiment are shown in Table S1. Details of statistics results are shown in Table S2.

To illustrate the effect of age on DA neurons in the SN, numbers of nigral neurons positively labeled by TH antibody were counted in 3-, 6-, 9-, and 12-month-old C57BL/6 mice. Results showed that overall TH^+^ signals in the SN decreased with age (Figure 1A). The number of TH^+^ neurons significantly decreased when mice reached 12 months old (Figure 1B). The DAT^+^ immunoreactive signals in the striatum of 9- and 12-month-old mice were lower than those of 3-month-old mice (Figure 1E,F).

We used Iba1 antibodies to label microglial cells and defined the degrees of microglial activation by the area (%) of Iba1^+^ immunoreactive signals and the number of Iba1^+^ cells in the sampled sections from the entire SN (Figure 1A). The area and number of Iba1^+^ cells in the SN were similar between the 3- and 6-month-old groups and gradually increased when they reached 9 and 12 months old (Figure 1C,D).

**Figure 2:**
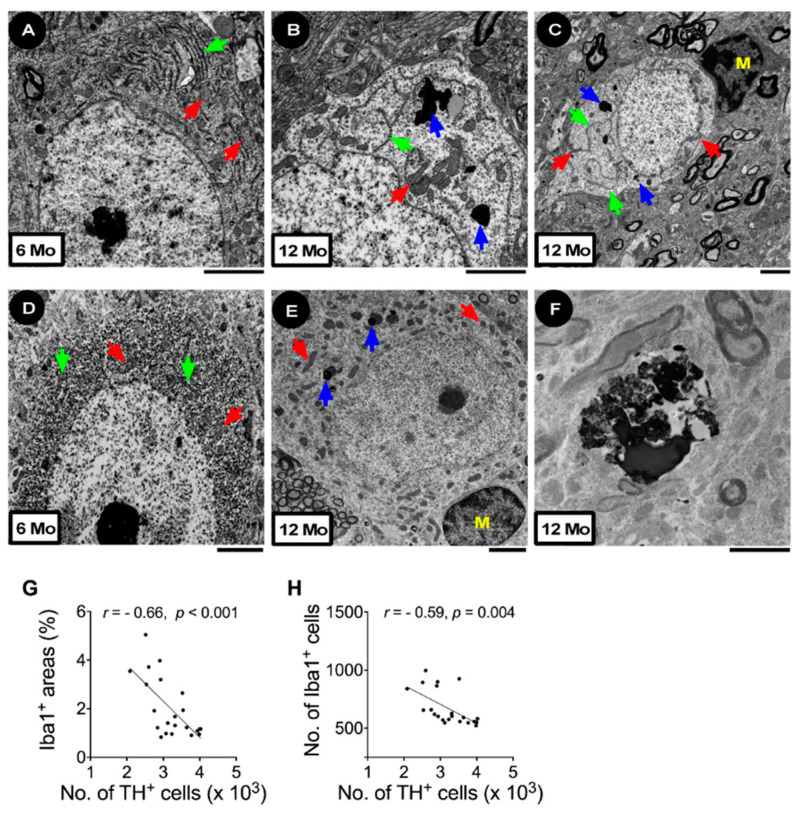
Association between the loss of DA neurons and microglial activation in the SN during aging. Representative EM (**A**–**C**) and TH^+^ immuno-EM (**D**–**F**) images of neurons in the SN of 6- and 12-month-old mice. (**A**) A neuron of a 6-month-old mouse. (**B**) A neuron of a 12-month-old mouse. (**C**) A microglia-like cell next to a neuron of a 12-month-old mouse. (**D**) A TH^+^ immuno-EM micrograph shows abundant TH^+^ granules in the cytoplasm of a 6-month-old mouse. (**E**) A TH^+^ neuron of a 12-month-old mouse. (**F**) A degenerating TH^+^ neuron of a 12-month-old mouse. Green arrows: rough endoplasmic reticulum; red arrows: mitochondria; blue arrows: lipofuscin-like electron-dense bodies; yellow M: microglia-like cells. Bar = 2 μm. (**G**) Pearson correlation between Iba1^+^ areas and the number of TH^+^ neurons. (**H**) Pearson correlation between the number of Iba1^+^ cells and the number of TH^+^ neurons. Each dot represents one mouse. The numbers of mice used in the experiment are shown in Table S1. Details of the statistical results are shown in Table S2.

The age-related loss of DA neurons was accompanied by motor impairment. In the beam traversal test, the time to traverse the beam (Figure 1G) and the number of foot faults (Figure 1H) were greater after the mice turned 9 months old. The tendency to fall in the rotarod test was also shorter after the mice were 9 months of age (Figure 1I). The age-related neurodegeneration in the SN was characterized by electron microscopy (EM). Images derived from EM (Figure 2A–C) and TH^+^ immuno-EM (Figure 2D–F) revealed that neurons in the SN of 6-month-old mice typically contained a centrally localized nucleus and organized rough endoplasmic reticulum (rER) (Figure 2A,D, green arrows) in the cytoplasm, with most mitochondria similar in size (Figure 2A,D, red arrows). In contrast, neurons in the SN of 12-month-old mice showed disordered organelles. Frequently, the rER became elongated and branched (Figure 2B,C, green arrows) and the mitochondria became inhomogeneous in size (Figure 2B,C,E, red arrows). Lipofuscin-like electron-dense bodies accumulated in the cytoplasm (Figure 2B,C,E, blue arrows). Microglia-like cells were frequently evident next to the degenerating neuron (Figure 2C,E, yellow M). TH^+^ signals were overtly and homogenously expressed in the neuron cytoplasm of 6-month-old mice (Figure 2D, small black granules), which were largely decreased in the neuron of 12-month-old mice (Figure 2E). There were also degenerating TH^+^ neurons, characterized by fragmented nuclei, darkened cytoplasm, membrane-encapsulated apoptotic bodies, and degenerating debris, in the SN of 12-month-old mice (Figure 2F).

The associations between the number of DA neurons and microglial activation were evaluated using the Pearson correlation. The area (Figure 2G) and number (Figure 2H) of Iba1^+^ cells were negatively correlated with the number of TH^+^ neurons in the SN.

### 3.2. Blocking Microglial Activation Suppresses Age-Related and Lipopolysaccharide-Induced DA Neuron Loss in the SN

**Figure 3:**
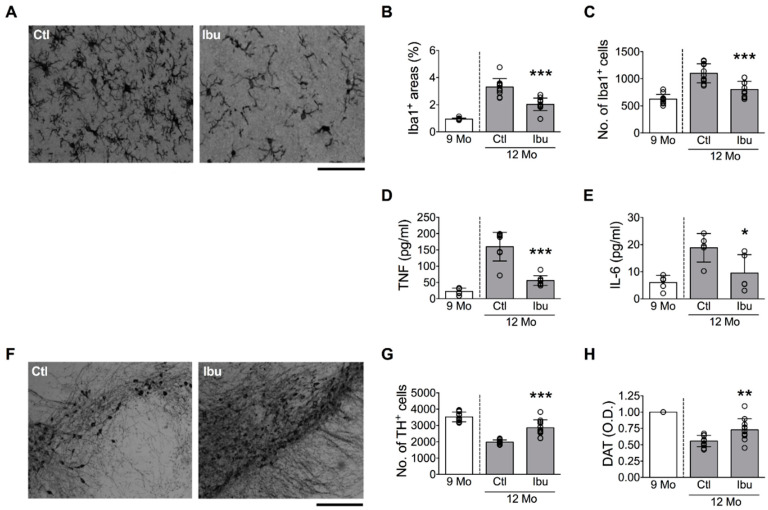
Suppression of age-related DA neuron loss in the SN by blocking microglial activation. Twelve-month-old mice were treated with (Ibu) or without (Ctl) ibuprofen for 3 months (from 9 to 12 months old). (**A**) Representative micrographs of Iba1^+^ cells in the SN. Bar = 50 μm. (**B**,**C**) Quantitative results of Iba1^+^ areas and numbers of Iba1^+^ cells. (**D**,**E**) Concentrations of TNF and IL-6 in the SN. (**F**) Representative micrographs of TH^+^ cells in the SN. Bar = 100 μm. (**G**) Quantitative results of TH^+^ cells in the SN. (**H**) Quantitative results of DAT^+^ signals in the striatum. The 9 Mo group was a reference group and was not included in the statistical analysis. * *p* < 0.05, ** *p* < 0.01, *** *p* < 0.001 versus Ctl group, unpaired two-tailed Student’s *t*-test. The numbers of mice used in the experiment are shown in Table S1. Details of statistics results are shown in Table S2.

To demonstrate the causal relationship between microglial activation and DA neuron loss in the SN, we performed two experiments. In the first experiment, 9-month-old mice were supplied with ibuprofen in the drinking water for 3 months to inhibit microglial activation. This age range was selected because overt microglial activation was detected in mice from 9 to 12 months old (Figure 1C,D). Results showed that ibuprofen significantly decreased the area and number of Iba1^+^ cells in the SN (Figure 3A–C), as well as local levels of TNF (Figure 3D) and IL-6 (Figure 3E). Significantly, ibuprofen treatment ameliorated the age-related loss of DA neurons in the SN (Figure 3F,G) and the decline of DAT^+^ signals in the striatum (Figure 3H).

**Figure 4:**
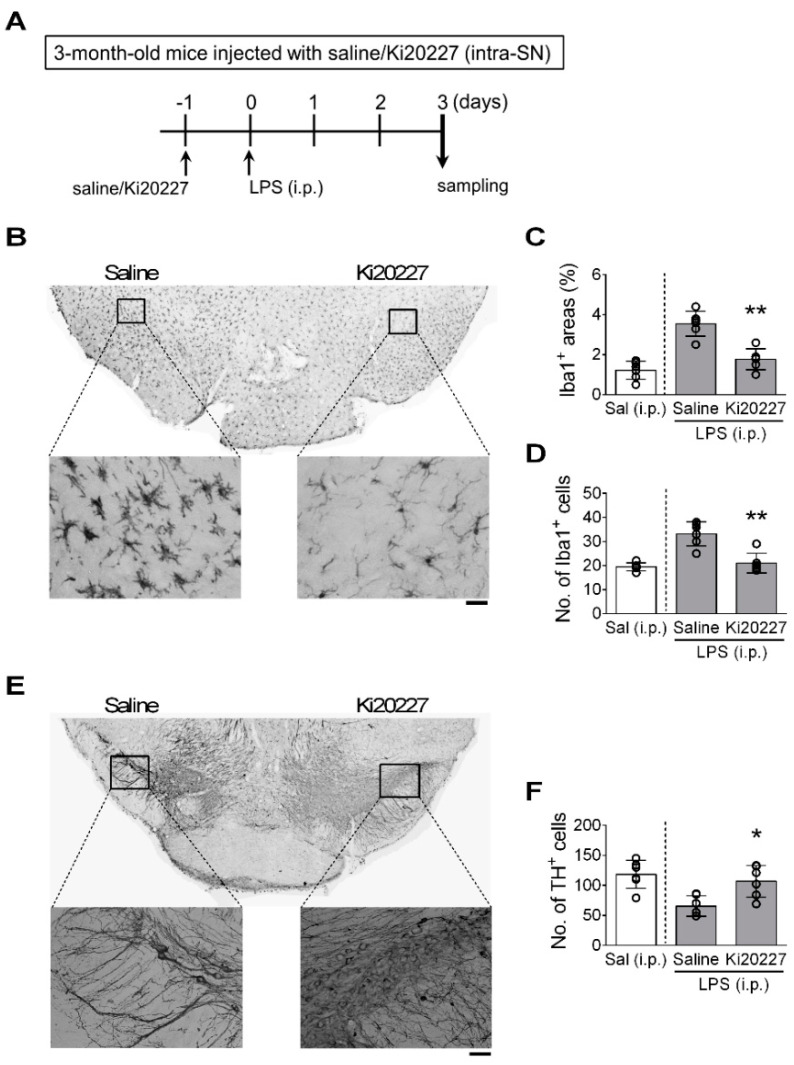
Decreasing the number of microglia in the SN inhibited LPS-induced DA neuron loss. (**A**) The experimental timeline. Three-month-old mice were injected with Ki20227 into their right SN and saline into their left SN one day before LPS injection (i.p.). Mice were sacrificed three days after the LPS injection. (**B**) Representative micrograph of Iba1^+^ cells in a mouse brain. The two boxed regions are enlarged. Bar = 50 μm. (**C**,**D**) Quantitative results of Iba1^+^ areas and numbers of Iba1^+^ cells. (**E**) Representative micrographs of TH^+^ cells in a mouse brain. Bar = 100 μm. (**F**) Quantitative results of TH^+^ cells in the SN. Sal (i.p.), a group of 3-month-old mice given an intraperitoneal injection of saline but no intra-SN injection, was a reference group and was not included in the statistical analysis. * *p* < 0.05, ** *p* < 0.01 versus saline injection side, paired two-tailed Student’s *t*-test. The numbers of mice used in the experiment are shown in Table S1. Details of the statistical results are shown in Table S2.

To demonstrate that microglial activation leads to DA neuron loss, in the second experiment, we injected LPS (i.p.) into 3-month-old mice to induce microglial activation as previously described [34]. One day before LPS injection, these mice had received an intra-SN injection of Ki20227, a selective colony-stimulating factor 1 receptor inhibitor that reduces microglia proliferation and viability [36], into their right SNs, while their left SNs received an equal volume of saline injection (Figure 4A). Results showed that three days after the LPS injection, the area and number of Iba1^+^ cells in the left SN (saline vehicle side) were increased (Figure 4B–D) with a reduced number of DA neurons (Figure 4E,F); however, in the right SN (Ki20227 side), the LPS-induced increases in the area and number of Iba1^+^ cells were significantly attenuated (Figure 4C,D) and the LPS-induced loss of TH^+^ cells was almost completely inhibited (Figure 4F). These results suggested that microglial activation is involved in DA neuron loss in the SN.

### 3.3. Running Exercise Ameliorates Age-Related Microglial Activation and DA Neuron

**Figure 5:**
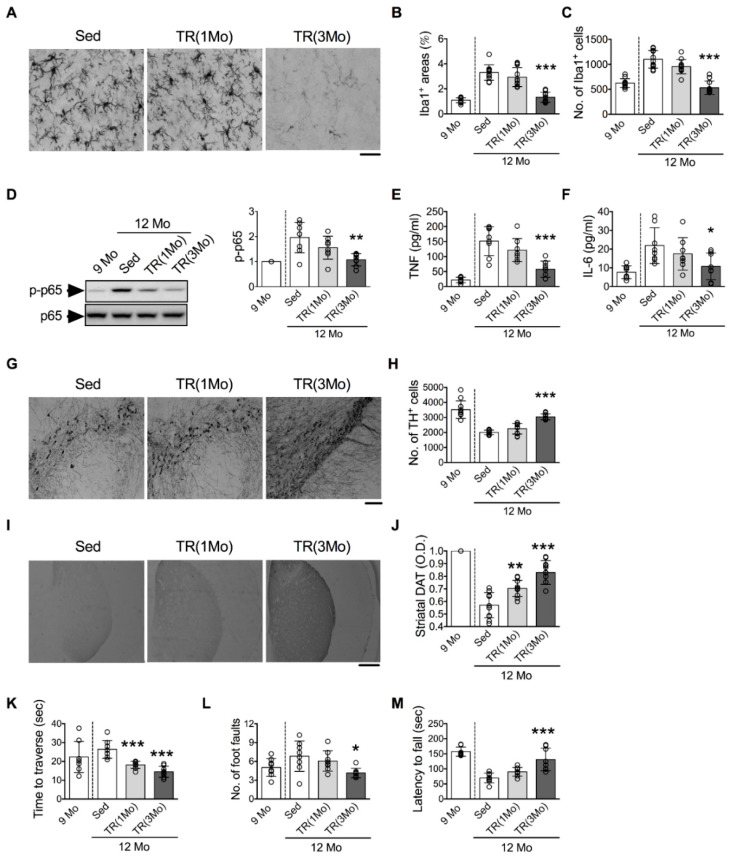
TR decreased age-related microglial activation and DA neuron loss in the SN, DAT^+^ reduction in the striatum, and motor deficit of middle-aged mice. (**A**) Representative micrographs of Iba1^+^ cells. Bar = 50 μm. (**B**,**C**) Quantitative results of Iba1^+^ areas and numbers of Iba1^+^ cells. (**D**) Relative levels of p-p65. Left panels: representative Western blots; right panel: quantitative results. (**E**,**F**) Concentrations of TNF and IL-6. (**G**) Representative micrographs of TH^+^ cells. Bar = 100 μm. (**H**) Quantitative results of TH^+^ cells. (**I**) Representative micrographs of DAT^+^ signals in the striata. Bar = 500 μm. (**J**) Quantitative results of DAT^+^ signals. (**K**–**M**) Performance of motor function. (**K**) Time to traverse the beam. (**L**) The number of foot faults made while traversing the beam. (**M**) Tendency to fall off the rotarod. The 9 Mo group was a reference group and was not included in the statistical analysis. * *p* < 0.05, ** *p* < 0.01, *** *p* < 0.001 versus Sed group, one-way ANOVA, Bonferroni’s post hoc test. The numbers of mice used in the experiment are shown in Table S1. Details of statistical results are shown in Table S2. Full-length blots are shown in Figure S2.

The effects of running exercise on age-related microglial activation and DA neuron loss in the SN were examined. Mice at 11 and 9 months old were given 1 and 3 months, respectively, of TR until they reached 12 months old (termed TR(1Mo) and TR(3Mo) groups, respectively). The age-related increases in the area and number of Iba1^+^ cells in the SN were almost completely inhibited in the TR(3Mo) group, but only partially in the TR(1Mo) group (Figure 5A–C). Likewise, the age-related increases of phosphorylated p65 (p-p65, Figure 5D), TNF (Figure 5E), and IL-6 (Figure 5F) were also attenuated in the TR(3Mo) group. The age-related decreases in the number of nigral TH^+^ cells (Figure 5G,H) and the density of striatal DAT^+^ signals (Figure 5I,J) were inhibited in the TR(3Mo) group. Finally, age-related motor impairments were significantly ameliorated in the TR(3Mo) group, as indicated by the shorter time to traverse and fewer foot faults in the beam traversal test, and a longer delay in the tendency to fall in the rotarod test than those of the Sed group (Figure 5K–M).

### 3.4. Inhibition of BDNF-TrkB Signaling Blocked the TR-Induced Protection against Age-Related Microglial Activation, DA Neuron Loss, and Motor Deficit

**Figure 6:**
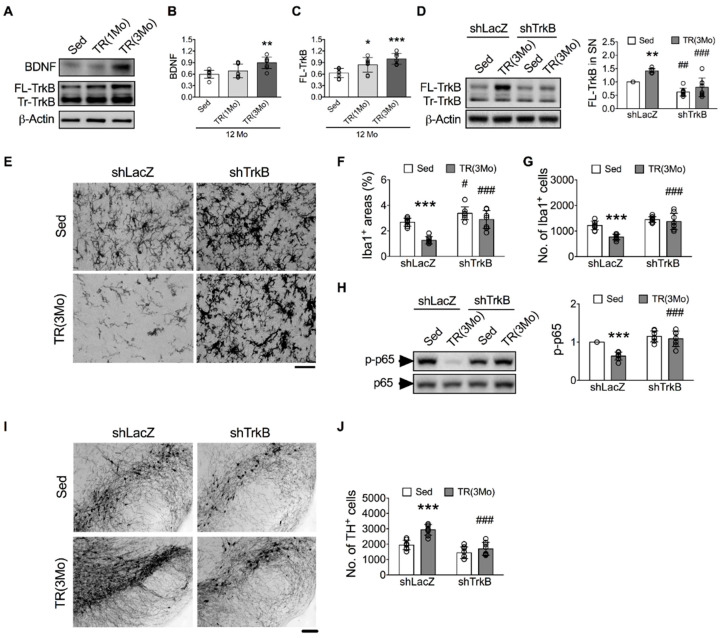
The downregulation of TrkB by shTrkB blocked the TR-induced inhibitions of age-related microglial activation and DA neuron loss in the SN. Mice were injected with shLacZ viruses into the left striata and shTrkB viruses into the right striata before and during (one injection each at 9-, 10-, and 11-month-old, respectively) the 3-month TR (TR(3Mo)). (**A**) Representative Western blots of BDNF and TrkB in the SN. (**B**,**C**) Quantitative results of BDNF and TrkB levels. * *p* < 0.05, ** *p* < 0.01, *** *p* < 0.001 versus Sed group, one-way ANOVA, Bonferroni’s post hoc test. (**D**) Relative levels of FL-TrkB. Left panels: representative Western blots; right panel: quantitative results. (**E**) Representative micrographs of Iba1^+^ cells in the SN. Bar = 20 μm. (**F**,**G**) Quantitative results of the areas and numbers of Iba1^+^ cells. (**H**) Relative levels of p-p65. Left panels: representative Western blots; right panel: quantitative results. (**I**) Representative micrographs of TH^+^ cells in the SN. Bar = 100 μm. (**J**) Quantitative results of TH^+^ cells. ** *p* < 0.01, *** *p* < 0.001 versus respective Sed group; # *p* < 0.05, ## *p* < 0.01, ### *p* < 0.001 versus respective shLacZ group, two-way ANOVA, Bonferroni’s post hoc test. The numbers of mice used in the experiment are shown in Table S1. Details of statistics results are shown in Table S2. Full-length blots are presented in Figure S2.

We investigated the role of the BDNF signaling pathway in TR-induced protection against age-related DA neuron loss because this pathway is enhanced by TR [12,34,48] and has been shown to inhibit microglial activation [43]. Compared to 12-month-old Sed mice, levels of BDNF (Figure 6B) and full-length TrkB (FL-TrkB, Figure 6C) were increased in the TR(3Mo) group, but in the TR(1Mo) group, only FL-TrkB levels were increased. The levels of truncated TrkB were also mildly affected in both TR groups (Tr-TrkB, Figure 6A).

The shTrkB treatment downregulated the levels of FL-TrkB in the SN (Figure 6D) and striatum (Figure S1A) of both Sed and TR(3Mo) mice. Importantly, the levels of FL-TrkB in the SN of TR(3Mo)-shTrkB group were comparable to those of Sed-shLacZ mice. The elimination of TR(3Mo)-induced increases in FL-TrkB levels completely blocked TR(3Mo)-induced inhibitions of age-related increases in the area and number of Iba1^+^ cells in the SN (Figure 6E-G) and striatum (Figure S1B,C), and levels of p-p65 in the SN (Figure 6H) and striatum (Figure S1D). Furthermore, the shTrkB treatment suppressed the TR(3Mo)-induced protection against age-related declines in the number of TH^+^ cells (Figure 6I,J) and the intensity of DAT^+^ signals (Figure S1E,F). Finally, the TR(3Mo)-induced protection against age-related motor deficit (tendency to fall in the rotarod test) was also suppressed by the shTrkB (Figure S1G).

## 4. Discussion

This study was designed to investigate the relationship between microglial activation and DA neuron loss in the SN during aging. We showed that the numbers of DA neurons were negatively correlated with degrees of microglial activation in the SN during aging. The microglial activation-related DA neuron death could be minimized by a long-term supplement of ibuprofen to decrease systemic inflammation and microglial activation in the SN, in agreement with previous observations that the inhibition of systemic and central inflammation by ibuprofen reduced DA neuron loss in PD animal models, as well as the risk of developing PD [49,50,51]. Furthermore, we demonstrated that microglial activation, caused by intraperitoneal LPS injection-induced systemic inflammation, led to DA neuron death in the SN, which could be blocked by eliminating microglial activation through intra-SN injection of Ki20227. These findings support a link between microglial activation and DA neuron death in the SN with the phenotypic consequence of motor impairment.

The fragmented nuclei and disordered organelles in the nigral DA neurons of 12-month-old mice were signs of degeneration. Lipofuscin granules and condensed apoptotic bodies were frequently evident in the DA neurons of middle-aged mice. Lipofuscin is a hallmark of dying neurons and is considered a precursor of neuronal death [1,52]. Interestingly, the inflammation-induced neuron loss in the SN was cell-type specific. The non-DA neuron subpopulations were less affected. In our estimation, the number of total neurons (i.e., NeuN^+^) in each side of the SN pars compacta was approximately 5000. Among them, about 4000 were DA neurons and 1000 were non-DA neurons. One week after LPS injection, the number of total neurons in the SN pars compacta reduced to about 4000, wherein 3000 were DA neurons and 1000 were non-DA neurons. The selective vulnerability of DA neurons has also been reported in aging non-human primates [1]. It appears that DA neurons are more vulnerable to inflammation-induced injury than are other types of neurons. Low levels of intracellular glutathione but high levels of dopamine, melanin, and lipids, all of which are prone to oxidation [1,53,54], render DA neurons highly susceptible to inflammatory insults.

It has been demonstrated that aging represents the most important risk factor for developing idiopathic PD, and DA neurons are preferentially vulnerable to aging [55,56]. The accumulated evidence indicates that the cause of age-related DA neuron loss in the SN is multifactorial [57]. These include oxidative stress [58,59,60,61], mitochondrial dysfunction [62,63,64], the impairment of protein clearance [65,66,67,68], and inflammation [1,69]. In this study, we focused on age-related systemic inflammation. It has been demonstrated that peripheral inflammatory signals can cross the blood-brain barrier and induce microglial activation [70], leading to the exaggerated synthesis of inflammatory cytokines and other mediators in the brain [71,72]. The age at which to see a significant DA neuron loss in C57BL mice varies among studies. In some studies, the number of DA neurons significantly decreased at either 9–12 months old [73,74,75] or 18–20 months old [76] in male C57BL mice. Our findings agree with the first group. However, some reports indicated no significant changes in the number of DA neurons up to 18 months old [77,78,79]. These differences in age-related DA deficits may be derived from different strains of C57BL mice (e.g., 6N or 6J) and/or housing conditions.

In addition to age, the male gender is also a known PD risk factor. It has been shown that age-related microglial activation and DA neuron loss were more pronounced in male mice than in females [40]. These differences could, at least partially, be attributed to the ovarian hormone, estrogen. The LPS-induced upregulations of Toll-like receptor 4, phosphorylated p38, and TNF in mouse BV2 microglial cells could be inhibited by the pretreatment of 17β-estradiol [40]. Likewise, an age-related decline in DA neuron firing activity was observed in 18-month-old male, but not female, mice [80]. This age-gender interaction was possibly linked to the PINK1- and PARK2-related adaptive response to age-related mitochondrial stress [80]. Therefore, only male mice were used in this study due to their higher degrees of microglial activation and DA neuron injury in the SN than those of female mice during aging.

The SN is one of the most sensitive brain regions to systemic inflammation [42,81]; this has been attributed to the high microglial density of the SN [42,82]. When encountering inflammatory signals, the regions with higher densities of microglia will accumulate higher levels of pro-inflammatory signals and, therefore, a higher degree of microglial activation. Because activated microglia chronically release neurotoxins [6], the brain regions with high densities of microglia are most vulnerable to inflammation.

Interestingly, Ki20227 has been shown to cause excessive microglial activation after cerebral ischemia, despite the blockage of microglial proliferation, resulting in increased neuronal degeneration [83]. The differential outcomes of Ki20227 in the cerebral ischemia model and our aging model may be due to different disease progressions. Physiologically, microglia play a vital role in maintaining the homeostasis of the brain by scavenging excess neurotoxins, removing cellular debris, and secreting neurotrophic factors [84,85]. In the ischemic brain, especially in the ischemic core, where cells die primarily due to necrosis, microglial phagocytic responses are vital in the removal of cell debris. Therefore, the elimination of microglia in the initial phase of ischemia may aggravate pathological progression [83]. However, in our aging model, Ki20227 eliminated microglia and related inflammatory responses; hence, it favored the survival of local DA neurons. Furthermore, it has been shown that in the ischemia model, the Ki20227 treatment polarized microglia toward the M1 phenotype, accompanied by increased concentrations of pro-inflammatory cytokines, such as IL-6 and TNF, and oxidative mediators [81], whereas the levels of anti-inflammatory cytokines, such as TGF-β, and antioxidant factors were decreased. Thus, microglia play an active and beneficial role in neuronal regeneration and functional recovery in the acute phase after injury [86]. However, chronic and hyperactivated microglia could induce secondary damage to intact tissue and inhibit post-injury repair.

In this study, we chose exercise training as a means of protecting DA neurons. The relationship between physical activities and PD has been investigated in a cohort of more than 210,000 participants [87]. According to participants’ physical activity intensities (i.e., light, moderate, and vigorous) at four age periods (i.e., 15–18, 19–29, and 35–39 years, and in the past 10 years before the diagnosis of PD), Xu et al. found that high levels of moderate to vigorous physical activities at ages 35–39 or in the past 10 years were dose-dependently associated with low PD risk. Individuals conducting moderate to vigorous physical activities in both periods had the highest protection (40% lower risk) than those who rarely performed moderate to vigorous exercise in both periods. Therefore, starting exercise in later life could still bring benefits in reducing the future risk of PD.

Long-term exercise increases the endogenous expression of BDNF and TrkB in the brain and enhances the proliferation of neural stem cells and neurite growth, as well as the survival of neuronal progenitor cells in the dentate gyrus [12,34,48]. It is well known that BDNF protects the brain from various insults. Most studies have attributed the neuroprotective effect of BDNF to the TrkB downstream pro-survival pathways [88,89]. Thus, even though BDNF has been reported to reduce inflammation in several brain injury models, the reduced inflammatory responses were generally considered a consequence of reduced neuron injury and death. Recently, the direct role of BDNF in regulating microglial activation has been demonstrated [43]. Through a series of pharmacological and genetic approaches in mouse and cultured cell models, Wu et al. showed that the expression level of TrkB in microglia determines the anti-microglial activation efficacy of BDNF [43]. Furthermore, the anti-microglial activation effect of BDNF involves the TrkB/Erk/CREB pathway. In line with these findings, we also found that reducing BDNF-TrkB signaling via shTrkB induced local microglial activation. Together, these results suggest that BDNF-TrkB signaling plays a critical role in modulating microglial activation during aging and represents an essential mechanism underlying the beneficial effects of exercise on brain function.

## 5. Conclusions

This study aimed to characterize the role of age-related microglial activation in the progressive DA neuron loss of aging mice. Initially, we found that the degrees of microglial activation were negatively correlated with the numbers of DA neuron loss in the SN of middle-aged mice, which loss causes impairments in motor balance and coordination function. Attenuating the effect of age-related microglial activation through pharmacological and exercise approaches, we found that the control of microglial activation reduced DA neuron loss in the SN of mice, indicating a pathogenic role of chronic microglial activation in PD. Furthermore, the running exercise regimen effectively inhibited microglial activation through the BDNF-TrkB signaling pathway. Exercise may serve as a non-pharmacological strategy to ameliorate neuroinflammation and aging-related neurodegenerative diseases.

## Data Availability

The data presented in this study are available in article and Supplementary Materials.

## Supplementary Materials

The following supporting information can be downloaded at: https://www.mdpi.com/article/10.3390/cells11030481/s1, Figure S1: Downregulation of TrkB with short hairpin (sh) TrkB blocked the treadmill-running (TR)-induced inhibition of age-related microglial activation and DAT^+^ reduction in the striatum and motor deficit of middle-aged mice; Figure S2: Full-length blots cropped for representative figures; Table S1: Sample sizes; Table S2: Details of statistical results.
