# Supplementary material for: Inhibition of Nigral Microglial Activation Reduces Age-Related Loss of Dopaminergic Neurons and Motor Deficits
Source: Cells. 2022 Jan 30;11(3):481. doi: 10.3390/cells11030481 (PMC8834087; doi:10.3390/cells11030481)
Supplement: Supplementary file 1 [file cells-11-00481-s001.zip › cells-1559372-supplementary.pdf]

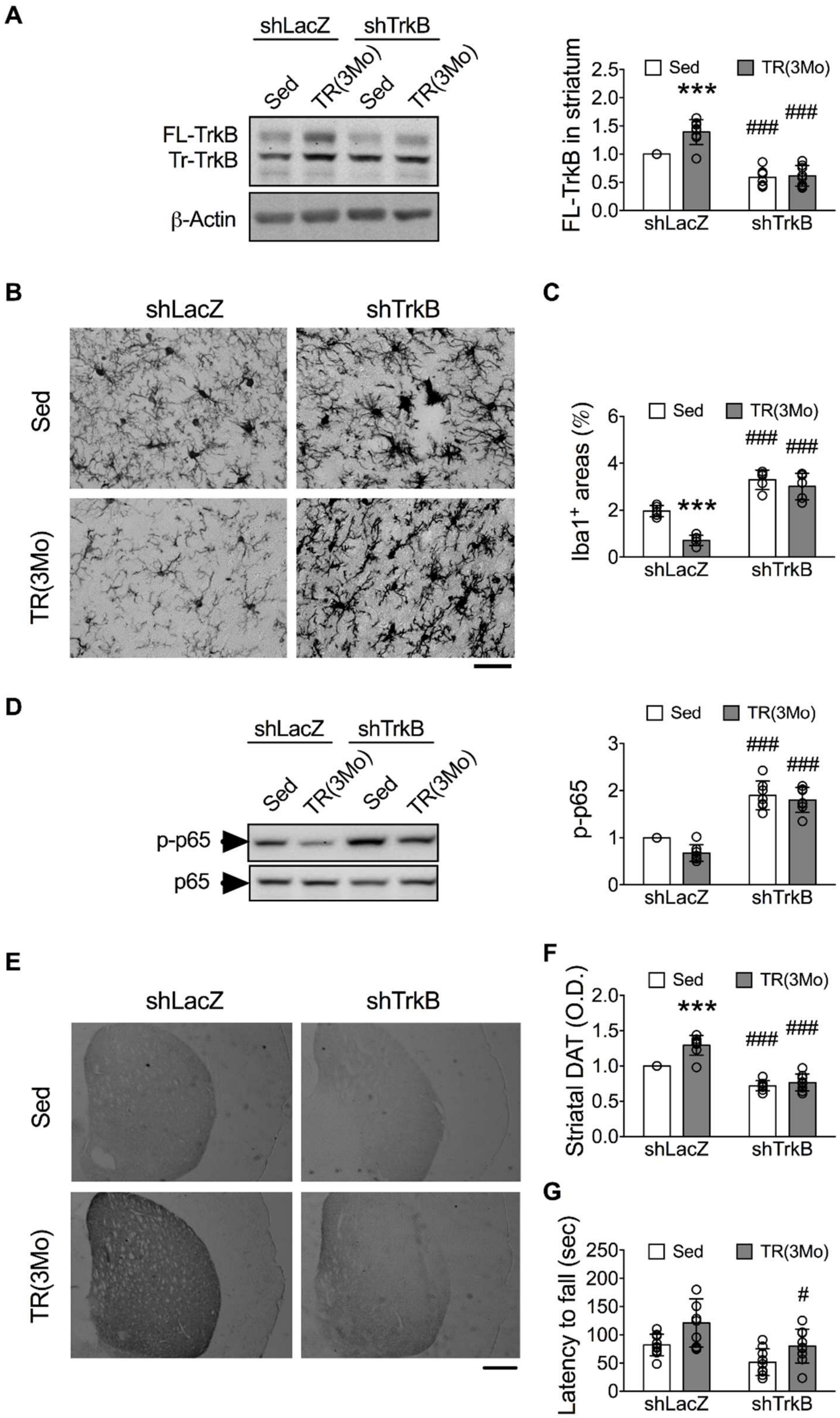

Figure S1. Downregulation of TrkB with short hairpin (sh)TrkB blocked the treadmill running (TR)-induced inhibition of age-related microglial activation and DAT reduction in the striatum and motor deficit of middle-aged mice. Mice that underwent 3 months of TR [TR(3Mo)] and three intrastriatal injections of shLacZ or shTrkB viruses. (A) Levels of full-length (FL)-TrkB. (B) Representative micrographs in the striata Bar = 20 μm. (C) Quantitative results of Iba1+ signals area. (D) Levels of p-p65 in the striata. (E) Representative micrographs of DAT+ signals in the striata. (F) Quantitative results of DAT+ signals. (G) Latency to fall off the rotarod. *** $p < 0.001$ versus respective Sed group; # $p < 0.05$, ### $p < 0.001$ versus respective shLacZ group, two-way ANOVA, Bonferroni's post hoc test. Full-length blots are presented in Figure S2.

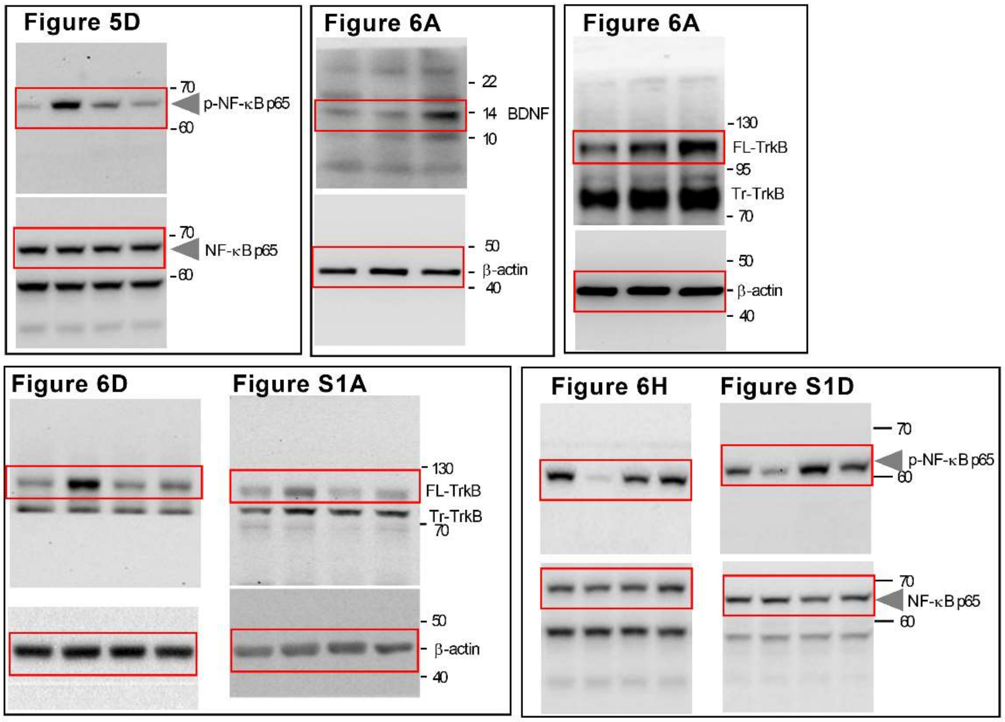

Figure S2. Full-length blots cropped for representative figures.

Table S1. Sample size

Panel	Groups	n
Figure 1B, C, D, F	3-month-old mice	6
	6-month-old mice	6
	9-month-old mice	5
	12-month-old mice	5
Figure 1G, H	6-month-old mice	8
	9-month-old mice	8
	12-month-old mice	8
Figure 1I	6-month-old mice	10
	9-month-old mice	10
	12-month-old mice	10
Figure 2G, H	Pearson correlation	22
Figure 3B, C, G, H	9 Mo	11
	Ctl	11
	Ibu	11
Figure 3D	9 Mo	8
	Ctl	8
	Ibu	8
Figure 3E	9 Mo	5
	Ctl	5
	Ibu	5
Figure 4C , D, F	Saline	6
	Ki20227	6
Figure 5B, C, H, J	9 Mo	11
	Sed	11
	TR(1Mo)	11
	TR(3Mo)	11
Figure 5D, E, F, K, L , M	9 Mo	8
	Sed	8
	TR(1Mo)	8
	TR(3Mo)	8
Figure 6B, C	Sed	8
	TR(1Mo)	8
	TR(3Mo)	8
Figure 6D, F,G, J, S1A, S1F	Sed-shLacZ	8
	TR-shLacZ	8
	Sed-shTrkB	8
	TR-shTrkB	8
Figure 6H, S1D	Sed-shLacZ	7
	TR-shLacZ	7
	Sed-shTrkB	7
	TR-shTrkB	7
Figure S1C	Sed-shLacZ	5
	TR-shLacZ	5
	Sed-shTrkB	5
	TR-shTrkB	5
Figure S1G	Sed-shLacZ	9
	TR-shLacZ	9
	Sed-shTrkB	9
	TR-shTrkB	9

Table S2. Details of statistics results

Statistics Methods and Results (part 1.)

Panel	Statistics methods	Statistics Results
Figure 1B	one-way ANOVA	F = 7.96, degree of freedom (d.f.) 3/18, $p = 0.001$
	Bonferroni's multiple comparisons test	3 Mo vs. 6 Mo: n.s.; 3 Mo vs. 9 Mo: n.s; 3 Mo vs. 12 Mo: $p = 0.006$
Figure 1C	one-way ANOVA	F = 19.53, *d.f.* 3/18, $p < 0.001$
	Bonferroni's multiple comparisons test	3 Mo vs. 6 Mo: n.s.; 3 Mo vs. 9 Mo: n.s; 3 Mo vs. 12 Mo: $p < 0.001$
Figure 1D	one-way ANOVA	F = 10.24, *d.f.* 3/18, $p < 0.001$
	Bonferroni's multiple comparisons test	3 Mo vs. 6 Mo: n.s.; 3 Mo vs. 9 Mo: $p = 0.009$; 3 Mo vs. 12 Mo: $p < 0.001$
Figure 1F	one-way ANOVA	F = 9.20, *d.f.* 3/18, $p < 0.001$
	Bonferroni's multiple comparisons test	3 Mo vs. 6 Mo: n.s.; 3 Mo vs. 9 Mo: $p = 0.009$; 3 Mo vs. 12 Mo: $p < 0.001$
Figure 1G	one-way ANOVA	F = 12.15, *d.f.* 2/21, $p < 0.001$
	Bonferroni's multiple comparisons test	6 Mo vs. 9 Mo: $p = 0.006$; 6 Mo vs. 12 Mo: $p < 0.001$
Figure 1H	one-way ANOVA	F = 31.53, *d.f.* 2/21, $p < 0.001$
	Bonferroni's multiple comparisons test	6 Mo vs. 9 Mo: $p < 0.001$; 6 Mo vs. 12 Mo: $p < 0.001$
Figure 1I	one-way ANOVA	F = 96.38, *d.f* . 2/27, $p < 0.001$
	Bonferroni's multiple comparisons test	6 Mo vs. 9 Mo: $p = 0.025$; 6 Mo vs. 12 Mo: $p < 0.001$
Figure 2G	Pearson correlation	TH neuron number v.s. Iba1 areas, $r = -0.66$, $p < 0.007$
Figure 2H	Pearson correlation	TH neuron number v.s. Iba1 cell numbers, $r = -0.59$, $p = 0.004$
Figure 3B	unpaired two-tailed Student's *t* test	t = 5.49, *d.f.* = 20, $p < 0.001$
Figure 3C	unpaired two-tailed Student's *t* test	t = 4.26, *d.f.* = 20, $p < 0.001$
Figure 3D	unpaired two-tailed Student's *t* test	t = 6.31, *d.f* . = 14, $p < 0.001$
Figure 3E	unpaired two-tailed Student's *t* test	t = 2.43, *d.f.* = 8, $p = 0.041$
Figure 3G	unpaired two-tailed Student's *t* test	t = 5.70, *d.f.* = 20, $p < 0.001$
Figure 3H	unpaired two-tailed Student's *t* test	t = 2.98, *d.f.* = 20, $p = 0.007$
Figure 4C	paired two-tailed Student's *t* test	t = 4.14, *d.f.* = 5, $p = 0.009$
Figure 4D	paired two-tailed Student's *t* test	t = 5.01, *d.f.* = 5, $p = 0.004$
Figure 4F	paired two-tailed Student's *t* test	t = 3.18, *d.f.* = 5, $p = 0.025$
Figure 5B	one-way ANOVA	F = 32.87, *d.f.* 2/30, $p < 0.001$
	Bonferroni's multiple comparisons test	Sed vs. TR(1Mo): n.s.; Sed vs. TR(3Mo): $p < 0.001$
Figure 5C	one-way ANOVA	F = 41.68, *d.f.* 2/30, $p < 0.001$
	Bonferroni's multiple comparisons test	Sed vs. TR(1Mo): n.s.; Sed vs. TR(3Mo): $p < 0.001$
Figure 5D	one-way ANOVA	F = 7.48, *d.f.* 2/21, $p = 0.004$
	Bonferroni's multiple comparisons test	Sed vs. TR(1Mo): n.s.; Sed vs. TR(3Mo): $p = 0.003$
Figure 5E	one-way ANOVA	F = 12.12, *d.f.* 2/21, $p < 0.001$
	Bonferroni's multiple comparisons test	Sed vs. TR(1Mo): n.s.; Sed vs. TR(3Mo): $p < 0.001$
Figure 5F	one-way ANOVA	F = 3.47, *d.f.* 2/21, $p = 0.050$
	Bonferroni's multiple comparisons test	Sed vs. TR(1Mo): n.s.; Sed vs. TR(3Mo): $p = 0.032$

Statistics Methods and Results (part 2.)		
**Panel**	**Statistics methods**	**Statistics Results**
Figure 5H	one-way ANOVA	F = 52.39, *d.f.* 2/30, $p < 0.001$
	Bonferroni's multiple comparisons test	Sed vs. TR(1Mo): n.s.; Sed vs. TR(3Mo): $p < 0.001$
Figure 5J	one-way ANOVA	F = 24.13, *d.f.* 2/30, $p < 0.001$
	Bonferroni's multiple comparisons test	Sed vs. TR(1Mo): $p = 0.004$; Sed vs. TR(3Mo): $p < 0.001$
Figure 5K	one-way ANOVA	F = 25.66, *d.f.* 2/21, $p < 0.001$
	Bonferroni's multiple comparisons test	Sed vs. TR(1Mo): $p < 0.001$; Sed vs. TR(3Mo): $p < 0.001$
Figure 5L	one-way ANOVA	F = 5.06, *d.f.* 2/21, $p = 0.016$
	Bonferroni's multiple comparisons test	Sed vs. TR(1Mo): n.s.; Sed vs. TR(3Mo): $p = 0.017$
Figure 5M	one-way ANOVA	F = 12.52, *d.f.* 2/21, $p < 0.001$
	Bonferroni's multiple comparisons test	Sed vs. TR(1Mo): n.s.; Sed vs. TR(3Mo): $p < 0.001$
Figure 6B	one-way ANOVA	F = 8.70, *d.f*. 2/21, $p = 0.002$
	Bonferroni's multiple comparisons test	Sed vs. TR(1Mo): n.s.; Sed vs. TR(3Mo): $p = 0.002$
Figure 6C	one-way ANOVA	F = 11.65, *d.f*. 2/21, $p < 0.001$
	Bonferroni's multiple comparisons test	Sed vs. TR(1Mo): $p = 0.036$; Sed vs. TR(3Mo): $p < 0.001$
Figure 6D	ordinary two-way ANOVA	interaction: F (1, 28) = 3.03, $p = 0.093$<br>shLacZ vs. shTrkB: F (1, 28) = 52.59, $p < 0.001$<br>Sed vs. TR: F (1, 28) = 18.83, $p < 0.001$
	Bonferroni's multiple comparisons test	shLacZ-TR(3Mo) vs. shLacZ-Sed: $p = 0.001$; shTrkB-Sed vs. shLacZ-Sed: $p = 0.003$; shTrkB-TR(3Mo) vs. shLacZ-TR(3Mo): $p < 0.001$; shTrkB-Sed vs. shTrkB-TR(3Mo): n.s.
Fgiure 6F	ordinary two-way ANOVA	interaction: F (1, 28) = 7.55, $p = 0.010$<br>shLacZ vs. shTrkB: F (1, 28) = 46.84, $p < 0.001$<br>Sed vs. TR: F (1, 28) = 31.22, $p < 0.001$
	Bonferroni's multiple comparisons test	shLacZ-TR(3Mo) vs. shLacZ-Sed: p < 0.001; shTrkB-Sed vs. shLacZ-Sed: p = 0.044; shTrkB-TR(3Mo) vs. shLacZ-TR(3Mo): p < 0.001; shTrkB-Sed vs. shTrkB-TR(3Mo): n.s.
Figure 6G	ordinary two-way ANOVA	interaction: F (1, 28) = 7.02 $p = 0.013$<br>shLacZ vs. shTrkB: F (1, 28) = 35.27, $p < 0.001$<br>Sed vs. TR: F (1, 28) = 14.29, $p < 0.001$
	Bonferroni's multiple comparisons test	shLacZ-TR(3Mo) vs. shLacZ-Sed: $p < 0.001$; shTrkB-Sed vs. shLacZ-Sed: n.s.; shTrkB-TR(3Mo) vs. shLacZ-TR(3Mo): $p < 0.001$; shTrkB-Sed vs. shTrkB-TR(3Mo): n.s.
Fgiure 6H	ordinary two-way ANOVA	interaction: F (1, 24) = 8.82, $p = 0.007$<br>shLacZ vs. shTrkB: F (1, 24) = 35.61, $p < 0.001$<br>Sed vs. TR: F (1, 24) = 18.00, $p < 0.001$
	Bonferroni's multiple comparisons test	shLacZ-TR(3Mo) vs. shLacZ-Sed: $p < 0.001$; shTrkB-Sed vs. shLacZ-Sed: n.s.; shTrkB-TR(3Mo) vs. shLacZ-TR(3Mo): $p < 0.001$; shTrkB-Sed vs. shTrkB-TR(3Mo): n.s.
Fgiure 6J	ordinary two-way ANOVA	interaction: F (1, 28) = 8.08, $p = 0.008$<br>shLacZ vs. shTrkB: F (1, 28) = 45.62, $p < 0.001$<br>Sed vs. TR: F (1, 28) = 23.47, $p < 0.001$
	Bonferroni's multiple comparisons test	shLacZ-Sed vs. shLacZ-TR(3Mo): $p < 0.001$; shLacZ-Sed vs. shTrkB-Sed: n.s.; shLacZ-TR(3Mo) vs. shTrkB-TR(3Mo): $p < 0.001$; shTrkB-Sed vs. shTrkB-TR(3Mo): n.s.

Statistics Methods and Results (part 3.)		
**Panel**	**Statistics methods**	**Statistics Results**
Figure S1A	ordinary two-way ANOVA	interaction: F (1, 28) = 9.96, *p* = 0.004<br>shLacZ vs. shTrkB: F (1, 28) = 106.82, *p* < 0.001<br>Sed vs. TR: F (1, 28) = 13.36, *p* = 0.001
	Bonferroni's multiple comparisons test	shLacZ-TR(3Mo) vs. shLacZ-Sed: *p* < 0.001; shTrkB-Sed vs. shLacZ-Sed: *p* < 0.001; shTrkB-TR(3Mo) vs. shLacZ-TR(3Mo): *p* < 0.001; shTrkB-Sed vs. shTrkB-TR(3Mo): n.s.
Fgiure S1C	ordinary two-way ANOVA	interaction: F (1, 16) = 7.88, *p* = 0.013<br>shLacZ vs. shTrkB: F (1, 16) = 110.37, *p* < 0.001<br>Sed vs. TR: F (1, 16) = 19.81, *p* < 0.001
	Bonferroni's multiple comparisons test	shLacZ-TR(3Mo) vs. shLacZ-Sed: *p* < 0.001; shTrkB-Sed vs. shLacZ-Sed: *p* < 0.001; shTrkB-TR(3Mo) vs. shLacZ-TR(3Mo): *p* < 0.001; shTrkB-Sed vs. shTrkB-TR(3Mo): n.s.
Fgiure S1D	ordinary two-way ANOVA	interaction: F (1, 24) = 1.88, *p* = 0.183<br>shLacZ vs. shTrkB: F (1, 24) = 148.40, *p* < 0.001<br>Sed vs. TR: F (1, 24) = 6.54, *p* = 0.017
	Bonferroni's multiple comparisons test	shLacZ-Sed vs. shLacZ-TR(3Mo): *p* = 0.0626; shLacZ-Sed vs. shTrkB-Sed: *p* < 0.001; shLacZ-TR(3Mo) vs. shTrkB-TR(3Mo): *p* < 0.001; shTrkB-Sed vs. shTrkB-TR(3Mo): n.s.
Fgiure S1F	ordinary two-way ANOVA	interaction: F (1, 28) = 12.60, *p* = 0.001<br>shLacZ vs. shTrkB: F (1, 28) = 134.07, *p* < 0.001<br>Sed vs. TR: F (1, 28) = 23.42, *p* < 0.001
	Bonferroni's multiple comparisons test	shLacZ-TR(3Mo) vs. shLacZ-Sed: *p* < 0.001; shTrkB-Sed vs. shLacZ-Sed: *p* < 0.001; shTrkB-TR(3Mo) vs. shLacZ-TR(3Mo): *p* < 0.001, shTrkB-TR(3Mo) vs. shTrkB-Sed: n.s.
Fgiure S1G	ordinary two-way ANOVA	interaction: F (1, 32) = 0.27, *p* = 0.605<br>shLacZ vs. shTrkB: F (1, 32) = 12.65, *p* = 0.001<br>Sed vs. TR: F (1, 32) = 11.34, *p* = 0.002
	Bonferroni's multiple comparisons test	shLacZ-TR(3Mo) vs. shLacZ-Sed: n.s.; shTrkB-Sed vs. shLacZ-Sed: n.s.; shTrkB-TR(3Mo) vs. shLacZ-TR(3Mo): *p* = 0.042, shTrkB-TR(3Mo) vs. shTrkB-Sed: n.s.
